# Supplementary material for: The reuse of public datasets in the life sciences: potential risks and rewards
Source: PeerJ. 2020 Sep 22;8:e9954. doi: 10.7717/peerj.9954 (PMC7518187; doi:10.7717/peerj.9954)
Supplement: Supplemental Information 1 [file peerj-08-9954-s001.pdf]

PMID	Title	First Author	Journal/Book	Publication Year	DOI
28480475	Clinical Data Reuse or Secondary Use: Current Status and Potential Future Progress	Meystre SM	Yearb Med Inform	2017	10.15265/IY-2017-007
26094821	Archetype-based data warehouse environment to enable the reuse of electronic health record data	Marco-Ruiz L	Int J Med Inform	2015	10.1016/j.ijmedinf.2015.05.016
30306916	Data Reuse Through Anesthesia Data Warehouse: Searching for New Use Contexts	Lamer A	Stud Health Technol Inform	2018	
29281658	Attitudes and norms affecting scientists' data reuse	Curty RG	PLoS One	2017	10.1371/journal.pone.0189288
26308551	Changes in Data Sharing and Data Reuse Practices and Perceptions among Scientists Worldwide	Tenopir C	PLoS One	2015	10.1371/journal.pone.0134826
29063535	Update on Data Reuse in Health Care	Safran C	Yearb Med Inform	2017	10.15265/IY-2017-013
31164773	Credit data generators for data reuse	Pierce HH	Nature	2019	10.1038/d41586-019-01715-4
24109559	Data reuse and the open data citation advantage	Piwowar HA	PeerJ	2013	10.7717/peerj.175
30961905	A review of attitudes towards the reuse of health data among people in the European Union: The primacy of purpose and the common good	Skovgaard LL	Health Policy	2019	10.1016/j.healthpol.2019.03.012
23269463	Reuse of public genome-wide gene expression data	Rung J	Nat Rev Genet	2013	10.1038/nrg3394
31426840	Clinical trial data reuse - overcoming complexities in trial design and data sharing	Wilkinson T	Trials	2019	10.1186/s13063-019-3627-6
28002728	Expanding the Immunology Toolbox: Embracing Public-Data Reuse and Crowdsourcing	Sparks R	Immunity	2016	10.1016/j.immuni.2016.12.008
30573830	Proteomics data reuse with MassIVE-KB	Doerr A	Nat Methods	2019	10.1038/s41592-018-0283-9
23508969	CistromeFinder for ChIP-seq and DNase-seq data reuse	Sun H	Bioinformatics	2013	10.1093/bioinformatics/btt135
29881734	A Data Quality Assessment Guideline for Electronic Health Record Data Reuse	Weiskopf NG	EGEMS (Wash DC)	2017	10.5334/egems.218
26769124	SPIRIT: Systematic Planning of Intelligent Reuse of Integrated Clinical Routine Data. A Conceptual Best-practice Framework and Procedure Model	Hackl WO	Methods Inf Med	2016	10.3414/ME15-01-0045
29758095	Reuse of public, genome-wide, murine eosinophil expression data for hypotheses development	Grace JO	J Leukoc Biol	2018	10.1002/JLB.1MA1117-444R
30895574	Impact of Electronic versus Paper-Based Recording before EHR Implementation on Health Care Professionals' Perceptions of EHR Use, Data Quality, and Data Reuse	Joukes E	Appl Clin Inform	2019	10.1055/s-0039-1681054
32160189	Data sharing, management, use, and reuse: Practices and perceptions of scientists worldwide	Tenopir C	PLoS One	2020	10.1371/journal.pone.0229003
31594947	Compendiums of cancer transcriptomes for machine learning applications	Lim SB	Sci Data	2019	10.1038/s41597-019-0207-2
24395549	Drug risk assessment and data reuse	Iyasu S	Pharmacoepidemiol Drug Saf	2014	10.1002/pds.3518
26137488	Toward a Literature-Driven Definition of Big Data in Healthcare	Baro E	Biomed Res Int	2015	10.1155/2015/639021
30342679	Kinematics of Big Biomedical Data to characterize temporal variability and seasonality of data repositories: Functional Data Analysis of data temporal evolution over non-parametric statistical manifolds	S√°ez C	Int J Med Inform	2018	10.1016/j.ijmedinf.2018.09.015
27374120	The harmonizome: a collection of processed datasets gathered to serve and mine knowledge about genes and proteins	Rouillard AD	Database (Oxford)	2016	10.1093/database/baw100
29677919	From Data Extraction to Analysis: Proposal of a Methodology to Optimize Hospital Data Reuse Process	Lamer A	Stud Health Technol Inform	2018	
22733976	Methods and dimensions of electronic health record data quality assessment: enabling reuse for clinical research	Weiskopf NG	J Am Med Inform Assoc	2013	10.1136/amiajnl-2011-000681
30957010	Three controversies in health data science	Peek N	Int J Data Sci Anal	2018	10.1007/s41060-018-0109-y
28906267	Evidence-Based Guidelines for Interface Design for Data Entry in Electronic Health Records	Wilbanks BA	Comput Inform Nurs	2018	10.1097/CIN.0000000000000387
26612985	Data standards can boost metabolomics research, and if there is a will, there is a way	Rocca-Serra P	Metabolomics	2016	10.1007/s11306-015-0879-3
26307996	High-Performance Motion Estimation for Image Sensors with Video Compression	Xu W	Sensors (Basel)	2015	10.3390/s150820752
30439700	Modern Information Technology for Cancer Research: What's in IT for Me? An Overview of Technologies and Approaches	Ahlbrandt J	Oncology	2018	10.1159/000493638
27170913	I-Maculaweb: A Tool to Support Data Reuse in Ophthalmology	Bonetto M	IEEE J Transl Eng Health Med	2015	10.1109/JTEHM.2015.2513043
29664470	Curated compendium of human transcriptional biomarker data	Golightly NP	Sci Data	2018	10.1038/sdata.2018.66
31956328	Regulating the Secondary Use of Data for Research: Arguments Against Genetic Exceptionalism	Martani A	Front Genet	2019	10.3389/fgene.2019.01254
29362097	Raising orphans from a metadata morass: A researcher's guide to re-use of public 'omics data	Bhandary P	Plant Sci	2018	10.1016/j.plantsci.2017.10.014
31314246	Research Data Stewardship for Healthcare Professionals	Jansen P	Fundamentals of Clinical Data Science	2019	10.1007/978-3-319-99713-1_4
29726424	A Clinical Data Warehouse Based on OMOP and i2b2 for Austrian Health Claims Data	Rinner C	Stud Health Technol Inform	2018	
30306898	Secondary Use of Healthcare Structured Data: The Challenge of Domain-Knowledge Based Extraction of Features	Chazard E	Stud Health Technol Inform	2018	
29298592	Linking temporal medical records using non-protected health information data	Bonomi L	Stat Methods Med Res	2018	10.1177/0962280217698005
30231853	AgriSeqDB: an online RNA-Seq database for functional studies of agriculturally relevant plant species	Robinson AJ	BMC Plant Biol	2018	10.1186/s12870-018-1406-2
28967481	Frameworks for Evaluating Medicines in Children	Turner MA	Clin Ther	2017	10.1016/j.clinthera.2017.09.003
26140212	Building a multi-scaled geospatial temporal ecology database from disparate data sources: fostering open science and data reuse	Soranno PA	Gigascience	2015	10.1186/s13742-015-0067-4
29796069	Barriers to the secondary use of data in critical care	Prince K	J Intensive Care Soc	2018	10.1177/1751143717741082
24534404	Building a common pediatric research terminology for accelerating child health research	Kahn MG	Pediatrics	2014	10.1542/peds.2013-1504
26267019	Neuroinformatics Software Applications Supporting Electronic Data Capture, Management, and Sharing for the Neuroimaging Community	Nichols BN	Neuropsychol Rev	2015	10.1007/s11065-015-9293-x
24625777	Refining gold from existing data	Wade TD	Curr Opin Allergy Clin Immunol	2014	10.1097/ACI.0000000000000051
31607804	Announcing the Journal of the Medical Library Association's data sharing policy	Akers KG	J Med Libr Assoc	2019	10.5195/jmla.2019.801
30612785	What can millions of laboratory test results tell us about the temporal aspect of data quality? Study of data spanning 17 years in a clinical data warehouse	Looten V	Comput Methods Programs Biomed	2019	10.1016/j.cmpb.2018.12.030
29557977	DASH, the data and specimen hub of the National Institute of Child Health and Human Development	Hazra R	Sci Data	2018	10.1038/sdata.2018.46
24395550	RE: Drug risk assessment and data reuse	Toh S	Pharmacoepidemiol Drug Saf	2014	10.1002/pds.3525
30794049	Data Dissemination: Shortening the Long Tail of Traumatic Brain Injury Dark Data	Hawkins BE	J Neurotrauma	2019	10.1089/neu.2018.6192
28637268	Predicting structured metadata from unstructured metadata	Posch L	Database (Oxford)	2016	10.1093/database/baw080
29346583	DataMed - an open source discovery index for finding biomedical datasets	Chen X	J Am Med Inform Assoc	2018	10.1093/jamia/ocx121
31550214	Evaluation of Research Accessibility and Data Elements of HIV Registries	Mayer CS	Curr HIV Res	2019	10.2174/1570162X17666190924195439
32102694	Large-scale public data reuse to model immunotherapy response and resistance	Fu J	Genome Med	2020	10.1186/s13073-020-0721-z
31437900	Interactive Machine Learning for Laboratory Data Integration	Fillmore N	Stud Health Technol Inform	2019	10.3233/SHTI190198
24245661	Impacts of structuring nursing records: a systematic review	Saranto K	Scand J Caring Sci	2014	10.1111/scs.12094
27899630	European Nucleotide Archive in 2016	Toribio AL	Nucleic Acids Res	2017	10.1093/nar/gkw1106
30306905	Comparison of Changes in the Number of Included Patients Between Interventional Trials and Observational Studies Published from 1995 to 2014 in Three Leading Journals	Dezetree A	Stud Health Technol Inform	2018	
18998885	Envisioning a biomedical data reuse registry	Piwowar HA	AMIA Annu Symp Proc	2008	
27570508	Data Citation in Neuroimaging: Proposed Best Practices for Data Identification and Attribution	Honor LB	Front Neuroinform	2016	10.3389/fninf.2016.00034
28703222	Transparency of genetic testing services for 'health, wellness and lifestyle': analysis of online prepurchase information for UK consumers	Hall JA	Eur J Hum Genet	2017	10.1038/ejhg.2017.75
24565381	Advancing data reuse in phyloinformatics using an ontology-driven Semantic Web approach	Panahiazar M	BMC Med Genomics	2013	10.1186/1755-8794-6-S3-S5
31409798	PlatformTM, a standards-based data custodianship platform for translational medicine research	Emam I	Sci Data	2019	10.1038/s41597-019-0156-9
30300549	Individual Variability of Protein Expression in Human Tissues	Kushner IK	J Proteome Res	2018	10.1021/acs.jproteome.8b00580
28881939	Data Management Rubric for Video Data in Organismal Biology	Brainerd EL	Integr Comp Biol	2017	10.1093/icb/icx060
29383564	Large Scale Analyses and Visualization of Adaptive Amino Acid Changes Projects	V√°zquez N	Interdiscip Sci	2018	10.1007/s12539-018-0282-7
25415993	Big heart data: advancing health informatics through data sharing in cardiovascular imaging	Suinesiaputra A	IEEE J Biomed Health Inform	2015	10.1109/JBHI.2014.2370952
28059691	Establishing data-intensive healthcare: the case of Hospital Electronic Prescribing and Medicines Administration systems in Scotland	Cresswell K	J Innov Health Inform	2016	10.14236/jhi.v23i3.842
31483250	Designing a Framework of Components to Support Patient Engagement in Research	√ñfelein M	Stud Health Technol Inform	2019	10.3233/SHTI190800
29043940	Using Maps to Promote Data-Driven Decision-Making: One Library's Experience in Data Visualization Instruction	Catalano MM	Med Ref Serv Q	2017	10.1080/02763869.2017.1369292
31984369	A tale of three subspecialties: Diagnosis recording patterns are internally consistent but Specialty-Dependent	Diaz-Garelli JF	JAMIA Open	2019	10.1093/jamiaopen/ooz020
30157511	On the Way to Close the Loop in Information Logistics: Data from the Patient - Value for the Patient	Hackl WO	Yearb Med Inform	2018	10.1055/s-0038-1667076
18769183	The first step toward data reuse: disambiguating concept representation of the locally developed ICU nursing flowsheets	Kim H	Comput Inform Nurs	2008	10.1097/01.NCN.0000304839.59831.28
28455596	Clinical decision support systems in child and adolescent psychiatry: a systematic review	Koposov R	Eur Child Adolesc Psychiatry	2017	10.1007/s00787-017-0992-0
30951089	An Integrated Genotyping-by-Sequencing Polymorphism Map for Over 10,000 Sorghum Genotypes	Hu Z	Plant Genome	2019	10.3835/plantgenome2018.06.0044
31315750	Constructing Large Scale Cohort for Clinical Study on Heart Failure with Electronic Health Record in Regional Healthcare Platform: Challenges and Strategies in Data Reuse	Liu DW	Chin Med Sci J	2019	10.24920/003579
30942868	Digital expression explorer 2: a repository of uniformly processed RNA sequencing data	Ziemann M	Gigascience	2019	10.1093/gigascience/giz022
22255972	Cubic spline interpolation with overlapped window and data reuse for on-line Hilbert Huang transform biomedical microprocessor	Chang NF	Conf Proc IEEE Eng Med Biol Soc	2011	10.1109/IEMBS.2011.6091792
26112029	The center for expanded data annotation and retrieval	Musen MA	J Am Med Inform Assoc	2015	10.1093/jamia/ocv048
22447219	Towards a human proteomics atlas	Gonnelli G	Anal Bioanal Chem	2012	10.1007/s00216-012-5940-8
32013947	Data Access Committees	Cheah PY	BMC Med Ethics	2020	10.1186/s12910-020-0453-z
31659323	A framework for tiered informed consent for health genomic research in Africa	Nembaware V	Nat Genet	2019	10.1038/s41588-019-0520-x
28423851	A Standardized and Data Quality Assessed Maternal-Child Care Integrated Data Repository for Research and Monitoring of Best Practices: A Pilot Project in Spain	S√°ez C	Stud Health Technol Inform	2017	
27092246	Patterns of database citation in articles and patents indicate long-term scientific and industry value of biological data resources	Bousfield D	F1000Res	2016	10.12688/f1000research.7911.1
21385382	Flow cytometry data standards	Spidlen J	BMC Res Notes	2011	10.1186/1756-0500-4-50
27693565	The Learning Healthcare System: Where are we now? A systematic review	Budrionis A	J Biomed Inform	2016	10.1016/j.jbi.2016.09.018
17917127	Sharing and reusing gene expression profiling data in neuroscience	Wan X	Neuroinformatics	2007	10.1007/s12021-007-0012-5
29295106	What Information Does Your EHR Contain? Automatic Generation of a Clinical Metadata Warehouse (CMDW) to Support Identification and Data Access Within Distributed Clinical Research Networks	Bruland P	Stud Health Technol Inform	2017	
27071884	Elderly Surgical Patients: Automated Computation of Healthcare Quality Indicators by Data Reuse of EHR	Ficheur G	Stud Health Technol Inform	2016	
26320941	Integration of extracellular RNA profiling data using metadata, biomedical ontologies and Linked Data technologies	Subramanian SL	J Extracell Vesicles	2015	10.3402/jev.v4.27497
27107447	Applying probabilistic temporal and multisite data quality control methods to a public health mortality registry in Spain: a systematic approach to quality control of repositories	S√°ez C	J Am Med Inform Assoc	2016	10.1093/jamia/ocw010
31336239	Applications of Fe(3)O(4)@AC nanoparticles for dye removal from simulated wastewater	Joshi S	Chemosphere	2019	10.1016/j.chemosphere.2019.07.011
22958225	Open by default: a proposed copyright license and waiver agreement for open access research and data in peer-reviewed journals	Hrynaszkiewicz I	BMC Res Notes	2012	10.1186/1756-0500-5-494
30523506	Lost in Datafication? - A Typology of (Emotion) Data Contextualization	Lehmann J	Integr Psychol Behav Sci	2019	10.1007/s12124-018-9470-6
21888989	Integrating clinical research with the Healthcare Enterprise: from the RE-USE project to the EHR4CR platform	El Fadly A	J Biomed Inform	2011	10.1016/j.jbi.2011.07.007
28637310	The metagenomic data life-cycle: standards and best practices	Ten Hoopen P	Gigascience	2017	10.1093/gigascience/gix047
25848584	The HMO Research Network Virtual Data Warehouse: A Public Data Model to Support Collaboration	Ross TR	EGEMS (Wash DC)	2014	10.13063/2327-9214.1049
28649160	Photon-HDF5: Open Data Format and Computational Tools for Timestamp-based Single-Molecule Experiments	Ingargiola A	Proc SPIE Int Soc Opt Eng	2016	10.1117/12.2212085
30147051	Learning Health Systems and Laboratory Medicine	Bietenbeck A	Stud Health Technol Inform	2018	
31852115	The number of stents was an independent risk of stent restenosis in patients undergoing percutaneous coronary intervention	Tang L	Medicine (Baltimore)	2019	10.1097/MD.0000000000018312
22865672	Two open access, high-quality datasets from anesthetic records	Cumin D	J Am Med Inform Assoc	2013	10.1136/amiajnl-2012-001087
26395036	Combining Health Data Uses to Ignite Health System Learning	Ainsworth J	Methods Inf Med	2015	10.3414/ME15-01-0064
25991173	Evaluation of compliance with recommendations of prevention of thromboembolism in atrial fibrillation in the elderly, by data reuse of electronic health records	Ferret L	Stud Health Technol Inform	2015	
21538885	A posteriori quality control for the curation and reuse of public proteomics data	Foster JM	Proteomics	2011	10.1002/pmic.201000602
28480704	Unbiased Protein Association Study on the Public Human Proteome Reveals Biological Connections between Co-Occurring Protein Pairs	Gupta S	J Proteome Res	2017	10.1021/acs.jproteome.6b01066
19592855	Towards a standardized representation to support data reuse: representing the ICNP semantics using the HL7 RIM	Kim H	Stud Health Technol Inform	2009	
28594819	Wide-Open: Accelerating public data release by automating detection of overdue datasets	Grechkin M	PLoS Biol	2017	10.1371/journal.pbio.2002477
28479569	A Web Based Tool to Enhance Monitoring and Retention in Care for Tuberculosis Affected Patients	Giannini B	Stud Health Technol Inform	2017	
27846806	GEN3VA: aggregation and analysis of gene expression signatures from related studies	Gundersen GW	BMC Bioinformatics	2016	10.1186/s12859-016-1321-1
23952476	Electronic health records: new opportunities for clinical research	Coorevits P	J Intern Med	2013	10.1111/joim.12119
27014709	Data Extraction and Management in Networks of Observational Health Care Databases for Scientific Research: A Comparison of EU-ADR, OMOP, Mini-Sentinel and MATRICE Strategies	Gini R	EGEMS (Wash DC)	2016	10.13063/2327-9214.1189
30919398	An openEHR Approach to Detailed Clinical Model Development: Tobacco Smoking Summary Archetype as a Case Study	Wei PC	Appl Clin Inform	2019	10.1055/s-0039-1681074
29192569	[From record keeping to scientific research: obstacles and opportunities for research with electronic health records]	Scholte RA	Ned Tijdschr Geneeskd	2017	
19151882	Perspectives for medical informatics. Reusing the electronic medical record for clinical research	Prokosch HU	Methods Inf Med	2009	
31390350	Temporal variability analysis reveals biases in electronic health records due to hospital process reengineering interventions over seven years	P√©rez-Benito FJ	PLoS One	2019	10.1371/journal.pone.0220369
27332243	Testing of Triggers by Data Mining of Epilepsy Patients' Structured Nursing Records	Kinnunen UM	Stud Health Technol Inform	2016	
23304327	ClinData Express--a metadata driven clinical research data management system for secondary use of clinical data	Li Z	AMIA Annu Symp Proc	2012	
28118921	Reusability of coded data in the primary care electronic medical record: A dynamic cohort study concerning cancer diagnoses	Sollie A	Int J Med Inform	2017	10.1016/j.ijmedinf.2016.08.004
23633602	Integrated database of information from structural genomics experiments	Asada Y	Acta Crystallogr D Biol Crystallogr	2013	10.1107/S0907444913001728
27529152	Quantitative monitoring of Arabidopsis thaliana growth and development using high-throughput plant phenotyping	Arend D	Sci Data	2016	10.1038/sdata.2016.55
24747879	The need for harmonized structured documentation and chances of secondary use - results of a systematic analysis with automated form comparison for prostate and breast cancer	Krumm R	J Biomed Inform	2014	10.1016/j.jbi.2014.04.008
23244533	Semantically enabling a genome-wide association study database	Beck T	J Biomed Semantics	2012	10.1186/2041-1480-3-9
24018520	Clinical professional governance for detailed clinical models	Goossen W	Stud Health Technol Inform	2013	
25848563	Recommendations for the use of operational electronic health record data in comparative effectiveness research	Hersh WR	EGEMS (Wash DC)	2013	10.13063/2327-9214.1018
25091808	Stability metrics for multi-source biomedical data based on simplicial projections from probability distribution distances	S√°ez C	Stat Methods Med Res	2017	10.1177/0962280214545122
31438173	SmartCRF: A Prototype to Visualize, Search and Annotate an Electronic Health Record from an i2b2 Clinical Data Warehouse	Cossin S	Stud Health Technol Inform	2019	10.3233/SHTI190476
32031623	The Data Tags Suite (DATS) model for discovering data access and use requirements	Alter G	Gigascience	2020	10.1093/gigascience/giz165
26152952	A Query Tool Enabling Clinicians and Researchers to Explore Patient Cohorts	Lim Choi Keung SN	Stud Health Technol Inform	2015	
28018995	Evaluating the Paper-to-Screen Translation of Participant-Aided Sociograms with High-Risk Participants	Hogan B	Proc SIGCHI Conf Hum Factor Comput Syst	2016	10.1145/2858036.2858368
29466022	From Liguria HIV Web to Liguria Infectious Diseases Network: How a Digital Platform Improved Doctors' Work and Patients' Care	Giannini B	AIDS Res Hum Retroviruses	2018	10.1089/aid.2017.0064
28625880	Predicting biomedical metadata in CEDAR: A study of Gene Expression Omnibus (GEO)	Panahiazar M	J Biomed Inform	2017	10.1016/j.jbi.2017.06.017
28049465	Secure and scalable deduplication of horizontally partitioned health data for privacy-preserving distributed statistical computation	Yigzaw KY	BMC Med Inform Decis Mak	2017	10.1186/s12911-016-0389-x
31029262	EMIF Catalogue: A collaborative platform for sharing and reusing biomedical data	Oliveira JL	Int J Med Inform	2019	10.1016/j.ijmedinf.2019.02.006
28974262	A generic method for improving the spatial interoperability of medical and ecological databases	Ghenassia A	Int J Health Geogr	2017	10.1186/s12942-017-0109-5
26306235	Rediscovering drug side effects: the impact of analytical assumptions on the detection of associations in EHR data	Diaz-Garelli JF	AMIA Jt Summits Transl Sci Proc	2015	
26538343	Discussion of "Combining Health Data Uses to Ignite Health System Learning"	Denaxas S	Methods Inf Med	2015	10.3414/ME15-12-0004
24996874	A patient-centered longitudinal care plan: vision versus reality	Dykes PC	J Am Med Inform Assoc	2014	10.1136/amiajnl-2013-002454
21262390	A comparison of two methods for retrieving ICD-9-CM data: the effect of using an ontology-based method for handling terminology changes	Yu AC	J Biomed Inform	2011	10.1016/j.jbi.2011.01.005
24302285	Data governance requirements for distributed clinical research networks: triangulating perspectives of diverse stakeholders	Kim KK	J Am Med Inform Assoc	2014	10.1136/amiajnl-2013-002308
21859004	A fast forward projection using multithreads for multirays on GPUs in medical image reconstruction	Chou CY	Med Phys	2011	10.1118/1.3591994
25065034	The promotion of data sharing in pharmacoepidemiology	Sethi N	Eur J Health Law	2014	10.1163/15718093-12341323
23201637	Harmonization and semantic annotation of data dictionaries from the Pharmacogenomics Research Network: a case study	Zhu Q	J Biomed Inform	2013	10.1016/j.jbi.2012.11.004
27071200	Embedded Streaming Deep Neural Networks Accelerator With Applications	Dundar A	IEEE Trans Neural Netw Learn Syst	2017	10.1109/TNNLS.2016.2545298
25150250	ProtocolNavigator: emulation-based software for the design, documentation and reproduction biological experiments	Khan IA	Bioinformatics	2014	10.1093/bioinformatics/btu554
27803687	Emerging Putative Associations between Non-Coding RNAs and Protein-Coding Genes in Neuropathic Pain: Added Value from Reusing Microarray Data	Raju HB	Front Neurol	2016	10.3389/fneur.2016.00168
26958300	Exploration of Temporal ICD Coding Bias Related to Acute Diabetic Conditions	McKillop M	AMIA Annu Symp Proc	2015	
32025475	The Argument Web: an Online Ecosystem of Tools, Systems and Services for Argumentation	Reed C	Philos Technol	2017	10.1007/s13347-017-0260-8
32151769	A content-based literature recommendation system for datasets to improve data reusability - A case study on Gene Expression Omnibus (GEO) datasets	Patra BG	J Biomed Inform	2020	10.1016/j.jbi.2020.103399
30122253	Earthquake prediction, biological clocks, and the cold war psy-ops: Using animals as seismic sensors in the 1970s California	Aronova E	Stud Hist Philos Sci	2018	10.1016/j.shpsa.2018.05.008
25653123	The importance of metadata to assess information content in digital reconstructions of neuronal morphology	Parekh R	Cell Tissue Res	2015	10.1007/s00441-014-2103-6
25000008	A Hybrid EAV-Relational Model for Consistent and Scalable Capture of Clinical Research Data	Khan O	Stud Health Technol Inform	2014	
31331268	JPhyloIO: a Java library for event-based reading and writing of different phylogenetic file formats through a common interface	St√∂ver BC	BMC Bioinformatics	2019	10.1186/s12859-019-2982-3
23793050	Knowledge management and informatics considerations for comparative effectiveness research: a case-driven exploration	Embi PJ	Med Care	2013	10.1097/MLR.0b013e31829b1de1
27376095	Performance of a Natural Language Processing (NLP) Tool to Extract Pulmonary Function Test (PFT) Reports from Structured and Semistructured Veteran Affairs (VA) Data	Sauer BC	EGEMS (Wash DC)	2016	10.13063/2327-9214.1217
31959844	Ontology-driven integrative analysis of omics data through Onassis	Galeota E	Sci Rep	2020	10.1038/s41598-020-57716-1
31424314	Higher body mass index is not a protective risk factor for 28-days mortality in critically ill patients with acute kidney injury undergoing continuous renal replacement therapy	Wang H	Ren Fail	2019	10.1080/0886022X.2019.1650767
18661966	SPECTRa: the deposition and validation of primary chemistry research data in digital repositories	Downing J	J Chem Inf Model	2008	10.1021/ci7004737
30595024	Evolution of Dementia Related to the Use of Alcohol in the French Nationwide Discharge Summary Database Between 2007 and 2017	Rochoy M	Am J Alzheimers Dis Other Demen	2019	10.1177/1533317518822043
24303283	Using clinical element models for pharmacogenomic study data standardization	Zhu Q	AMIA Jt Summits Transl Sci Proc	2013	
26998241	ISCB's initial reaction to New England Journal of Medicine editorial on data sharing	Valencia A	F1000Res	2016	10.12688/f1000research.8051.1
28876906	Water Issues Related to Transitioning from Conventional to Unconventional Oil Production in the Permian Basin	Scanlon BR	Environ Sci Technol	2017	10.1021/acs.est.7b02185
25463966	Using electronic health records for clinical research: the case of the EHR4CR project	De Moor G	J Biomed Inform	2015	10.1016/j.jbi.2014.10.006
24126951	[Implementation of a patient data management system. Effects on intensive care documentation]	Castellanos I	Anaesthesist	2013	10.1007/s00101-013-2239-x
14625855	Hierarchical analysis of large-scale two-dimensional gel electrophoresis experiments	Rubinfeld A	Proteomics	2003	10.1002/pmic.200300533
25848610	Sustainability considerations for health research and analytic data infrastructures	Wilcox A	EGEMS (Wash DC)	2014	10.13063/2327-9214.1113
27444186	Use of "off-the-shelf" information extraction algorithms in clinical informatics: A feasibility study of MetaMap annotation of Italian medical notes	Chiaramello E	J Biomed Inform	2016	10.1016/j.jbi.2016.07.017
30756117	Shift in Hospitalizations for Alzheimer's Disease to Related Dementias in France between 2007 and 2017	Rochoy M	J Prev Alzheimers Dis	2019	10.14283/jpad.2019.5
10144371	Information technology in the foxhole	Eyestone SM	Top Health Inf Manage	1995	
32171029	Enabling reusability of plant phenomic datasets with MIAPPE 1.1	Papoutsoglou EA	New Phytol	2020	10.1111/nph.16544
31040006	Open access image repositories: high-quality data to enable machine learning research	Prior F	Clin Radiol	2020	10.1016/j.crad.2019.04.002
21342582	FPGA accelerator for protein secondary structure prediction based on the GOR algorithm	Xia F	BMC Bioinformatics	2011	10.1186/1471-2105-12-S1-S5
21678495	AnatomicalTerms.info: heading for an online solution to the anatomical synonym problem hurdles in data-reuse from the Terminologia Anatomica and the foundational model of anatomy and potentials for future development	Gob√©e OP	Clin Anat	2011	10.1002/ca.21185
28466088	Combining Contrast Mining with Logistic Regression To Predict Healthcare Utilization in a Managed Care Population	Sheets L	Appl Clin Inform	2017	10.4338/ACI-2016-05-RA-0078
21893862	Recording associated disorders using SNOMED CT	Cornet R	Stud Health Technol Inform	2011	
20442144	International classification of diseases, 10th edition, clinical modification and procedure coding system: descriptive overview of the next generation HIPAA code sets	Steindel SJ	J Am Med Inform Assoc	2010	10.1136/jamia.2009.001230
32324446	Impact on Quality of Documentation and Workload of the Introduction of a National Information Standard for Tumor Board Reporting	Ebben KCWJ	JCO Clin Cancer Inform	2020	10.1200/CCI.19.00050
25428570	Empirical study using network of semantically related associations in bridging the knowledge gap	Abedi V	J Transl Med	2014	10.1186/s12967-014-0324-9
25967024	Region-referenced phase unwrapping architecture for digital holographic microscopy	Hwang WJ	Appl Opt	2015	10.1364/AO.54.000A67
32308882	De-identification of Clinical Text via Bi-LSTM-CRF with Neural Language Models	Tang B	AMIA Annu Symp Proc	2020	
25848591	Learning from health information exchange technical architecture and implementation in seven beacon communities	McCarthy DB	EGEMS (Wash DC)	2014	10.13063/2327-9214.1060
32301862	Research on the Radiotoxicology of Plutonium Using Animals: Consideration of the 3Rs-Replace, Reduce, Refine	Griffiths NM	Health Phys	2020	10.1097/HP.0000000000001258
25848582	Validation of Diagnostic and Procedural Codes for Identification of Acute Cardiovascular Events in US Veterans with Rheumatoid Arthritis	Davis LA	EGEMS (Wash DC)	2014	10.13063/2327-9214.1023
32521555	Factors Influencing Problem List Use in Electronic Health Records-Application of the Unified Theory of Acceptance and Use of Technology	Klappe ES	Appl Clin Inform	2020	10.1055/s-0040-1712466
21346966	Development of automated quality reporting: aligning local efforts with national standards	Dykes PC	AMIA Annu Symp Proc	2010	
24808228	Efficient acceleration of mutual information computation for nonrigid registration using CUDA	Ikeda K	IEEE J Biomed Health Inform	2014	10.1109/JBHI.2014.2310745
32418147	Development, implementation and preliminary evaluation of clinical dashboards in a department of anesthesia	Laurent G	J Clin Monit Comput	2020	10.1007/s10877-020-00522-x
19208138	Fine-grained parallel RNAalifold algorithm for RNA secondary structure prediction on FPGA	Xia F	BMC Bioinformatics	2009	10.1186/1471-2105-10-S1-S37
